# Importance of the relationship between symptoms and self-reported physical activity level in stable COPD based on the results from the SPACE study

**DOI:** 10.1186/s12931-019-1053-7

**Published:** 2019-05-14

**Authors:** Florin Mihaltan, Yochai Adir, Adam Antczak, Konstantinos Porpodis, Vesna Radulovic, Nuno Pires, Geeuwke Jan de Vries, Andreas Horner, Samuel De Bontridder, Yunqin Chen, Anat Shavit, Silviu Alecu, Lukasz Adamek

**Affiliations:** 1 Department of Pulmonology, University of Medicine and Pharmacy “Carol Davila”, Bucharest, Romania; 2 Faculty of Medicine, Technion-Israel Institute of Technology, Haifa, Israel; 3 Clinical Department of Pulmonology and Allergology, University Hospital, Lodz, Poland; 4 Thessaloniki University Medical School, Thessaloniki, Greece; 5 Municipal Institute for Lung Diseases and Tuberculosis, Belgrade, Serbia; 6 Hospital Santa Maria Maior, Barcelos, Portugal; 7 Zuyderland Ziekenhuis Sittard, Geleen, Netherlands; 8 Kepler University Hospital, Krankenhausstrasse 9, A4021, Linz, Austria; 9 UZ Brussel, Laarbeeklaan 101, 1090 Jette, Belgium; 10 AstraZeneca Global R&D Information, Shanghai, China; 11 AstraZeneca Germany, Wedel, Germany; 12 AstraZeneca Romania, Bucharest, Romania; 13 AstraZeneca Europe, Luton, UK

**Keywords:** COPD, GOLD, Physical activity, Sedentarism, SPACE, Symptoms

## Abstract

**Background:**

The burden of symptoms and risk of exacerbations are the main drivers of the overall assessment of the Chronic Obstructive Pulmonary Disease (COPD) and the adequate treatment approaches per current Global Initiative for Chronic Obstructive Lung Disease (GOLD). Physical activity has emerged as both functional outcome and non-pharmacological intervention in COPD patients, despite the lack of standardized measures or guidelines in clinical practice. This study aimed to explore in more depth the 24-h respiratory symptoms, the physical activity level (PAL) and the relationship between these two determinants in stable COPD patients.

**Methods:**

This was a multinational, multicenter, observational, cross-sectional study conducted in ten European countries and Israel. Dedicated questionnaires for each part of the day (morning, daytime, night) were used to assess respiratory symptoms. PAL was evaluated with self- and interview-reported tools [EVS (exercise as vital sign) and YPAS (Yale Physical Activity Survey)], and physician’s judgement. Patients were stratified in ABCD groups by 2013 and 2017 GOLD editions using the questionnaires currently recommended: modified Medical Research Council dyspnea scale and COPD Assessment Test.

**Results:**

The study enrolled 2190 patients (mean age: 66.9 years; male: 70.0%; mean % predicted FEV1: 52.6; GOLD groups II-III: 84.5%; any COPD treatment: 98.9%). Most patients (> 90%) reported symptoms in any part of the 24-h day, irrespective of COPD severity. PAL evaluations showed discordant results between patients and physicians: 32.9% of patients considered themselves completely inactive, while physicians judged 11.9% patients as inactive. By YPAS, the overall study population spent an average of 21.0 h/week performing physical activity, and 68.4% of patients were identified as sedentary. In any GOLD ABCD group, the percentage of inactive patients was high. Our study found negative, weak correlations between respiratory symptoms and self-reported PAL (*p* < 0.001).

**Conclusions:**

Despite regular treatment, the majority of stable COPD patients with moderate to severe disease experienced daily variable symptoms. Physical activity level was low in this COPD cohort, and yet overestimated by physicians. With evidence indicating the negative consequences of inactivity, its adequate screening, a more active promotion and regular assessment of physical activity are urgently needed in COPD patients for better outcomes.

**Trial registration:**

NCT03031769, retrospectively registered, 23 Jan 2017.

**Electronic supplementary material:**

The online version of this article (10.1186/s12931-019-1053-7) contains supplementary material, which is available to authorized users.

## Background

Chronic Obstructive Pulmonary Disease (COPD) is a common disorder associated with significant morbidity and mortality, despite its preventable and treatable nature [1]. In 2011, the Global Initiative for Obstructive Lung Disease (GOLD) [2] added symptom burden tothe multidimensional assessment, to classify the patients into four quadrants (GOLD ABCD), combining it with the risk of exacerbations based on pulmonary function and history of exacerbations. In 2017, the classification system was simplified, separating the spirometric measures from the evaluation of respiratory symptoms and exacerbation history [3].

The prevalence, intensity and variability of COPD symptoms have been extensively explored in the literature, demonstrating their significant impact on patients’ daily activities, health-related and overall quality of life [4–11]. Early morning, daytime and night-time symptoms have been investigated either separately [4, 8–11], or throughout the 24-h day [7, 12], showing negative associations with a broad range of patient-reported outcomes such as exacerbations, anxiety, depression, sleep and physical activity.

Physical activity (PA) has emerged as an important functional outcome in COPD patients, based on its association with mortality and exacerbations [13]. Consequently, increasing PA has become a significant non-pharmacological intervention in patients with COPD [1]. Physical activity is a complex multidimensional construct characterized by frequency, duration, intensity and type of activity, [14] and affected by individual, physiological, environmental and psycho-social factors [15, 16]. Recognizing the patients at risk for low physical activity is crucial for clinicians and although many methods are available for the assessment of PA, no measure or guidance is standardized [17–20].

In the light of recent changes in GOLD and the higher importance of PA in COPD evolution, a better understanding of the prevalence and severity of respiratory symptoms, physical activity level and the relationship between these two determinants of quality of life in real-life practice is needed. Therefore, the aims of our study were to explore in more depth the respiratory symptoms over a 24-h period in stable COPD patients, the patterns of self-reported physical activity and their relationship in various healthcare systems across Western and Central Eastern Europe and Israel.

## Methods

### Study population

Eligible patients were aged at least 40 years with an established COPD history (minimum 1 year since diagnosis). A post-bronchodilator forced expiratory volume in 1 s / forced vital capacity (FEV_1_/FVC) ratio of < 0.7 in the year before enrollment had to be documented. All patients were current or former smokers with a smoking history of at least 10 pack-years and stable disease (no exacerbation history or changes in current COPD maintenance treatment in the 2 months prior to enrollment). Exclusion criteria included: history of any chronic respiratory disease other than COPD or sleep apnea syndrome, any acute or chronic condition that would have limited the patients’ ability to complete the questionnaires, and concomitant enrollment in a clinical trial that would have influenced participation in the study. Patients with a previous diagnosis of asthma or non-idiopathic pulmonary fibrosis were included only if COPD was the main diagnosis. The enrollment started in December 2016 and lasted until August 2017.

### Study design

The Symptoms and Physical Activity in COPD patients in Europe (SPACE study, NCT03031769) was a multinational, multicenter, observational, cross-sectional study conducted in 144 centers across 11 countries (Austria, Belgium, Bulgaria, Greece, Israel, The Netherlands, Poland, Portugal, Romania, Serbia, Slovakia). Patients were recruited from pulmonology and primary care practices and hospitals. Patients with stable COPD who fulfilled the eligibility criteria were identified at each site and invited to participate in the study. To minimize selection bias, each site was allowed to enroll a maximum quota of patients as determined at country level. The study had a single visit; no interventions or extra procedures beyond clinical practice were required.

At the study visit, the investigators collected the following information: socio-demographics, comorbidities, year of COPD diagnosis, current COPD treatment, spirometry, number of exacerbations and COPD related visits to general practitioners and specialists in the last year. An exacerbation was defined as an acute worsening of respiratory symptoms that resulted in additional therapy, e.g., bronchodilators only (mild exacerbation), bronchodilators plus antibiotics and/or oral corticosteroids (moderate exacerbation), or requiring hospitalization or emergency room visits (severe exacerbation). Information was collected from medical charts and during interviews with patients. The COPD specific comorbidities test (COTE) index [21] and the combined index of body mass index, obstruction, dyspnea and exacerbations (BODEx) [22] were calculated. Patients were allocated into groups A to D following the GOLD 2013 [23] and GOLD 2017 recommendations [3].

The study and the informed consent form were approved by Institutional Review Boards or Ethics Committees in each participating country according to the local applicable legislation on observational studies. All patients provided their written informed consent before enrollment.

### Assessments

SPACE study participants completed several interviewer- and patient-administered questionnaires. Dyspnea was assessed based on the patient’s response according to the modified Medical Research Council (mMRC) scale [24] (from 0 = breathlessness with strenuous exercise to 4 = too breathless to leave the house or breathless when dressing or undressing). Health status and symptomatic impact of COPD was determined using the COPD Assessment Test (CAT) [25] with scores ranging from 0 to 40, where higher scores indicate a worse health status. The GOLD ABCD assessment tool considers either the cut-off of ≥2 for mMRC or ≥ 10 for CAT to separate the low- and high-symptom burden groups [3, 23].

The COPD symptoms (dyspnea, cough and sputum and chest symptoms) were evaluated using dedicated questionnaires for each part of the day [26–28]. The occurrence and severity of night-time symptoms, nocturnal awakenings and rescue medication use due to symptoms were assessed with the Night-time Symptoms of COPD Instrument (NiSCI) [26]. Daytime symptoms were evaluated using a derivative instrument based on the Evaluating Respiratory Symptoms in COPD (E-RS™: COPD) questionnaire, which quantified the respiratory symptoms in stable patients using total score (RS-Total score) and subscale scores (RS-Chest symptoms, RS-Cough & Sputum, RS-Breathlessness) [27]. Total score ranges were from 0 to 40, with higher scores indicating more severe symptoms. The occurrence and severity of early morning symptoms, rescue medication use and limitations of activities due to symptoms were assessed with the Early Morning Symptoms of COPD Instrument (EMSCI) [28]. For EMSCI and NiSCI, responses were coded from 0 to 4 (e.g., 0 = no symptoms, 1 = mild, 2 = moderate, 3 = severe, 4 = very severe). For night-time and early morning symptoms, two separate scores were calculated: an overall COPD severity domain score and a 6-item symptom severity score. Printed versions of the NiSCI and EMSCI questionnaires, customized for the SPACE study, were used to collect patient responses.

At the study visit, the investigators assessed the physical activity level of patients, according to their clinical judgement, and classified patients as active (performing ≥150 min/week of moderate to vigorous exercise, such as brisk walking), insufficiently active (performing 1–149 min/week of moderate to vigorous exercise) or completely inactive (0 min/week of moderate to vigorous exercise) [29]. Patients self-reported their level of physical activity by answering two questions during clinical interview. These questions were derived from the exercise vital sign (EVS) program [30] and refer to the number of days per week that patients engage in moderate to strenuous exercise and the number of minutes spent doing such exercise. Response choices were categorical and the electronic Case Report Form (CRF) automatically calculated the total minutes per week of moderate to vigorous physical activity, without displaying these responses to the investigator. The categories of physical activity level that were used in these questions were the same as those used in the investigator’s assessment. The physical activity of patients was also evaluated with the Yale Physical Activity Survey (YPAS) [31]. YPAS is a detailed interviewer-administrated questionnaire specifically developed for senior subjects and validated for use in COPD patients [32]. The questionnaire is divided into two parts, with a checklist to estimate the time spent in different groups of activities in a typical week from the past month and a section with questions assessing the participation in different types of activities (e.g., vigorous activity, sitting, standing) [31]. The summary index score, derived from the second part of the questionnaire, ranged from 0 to 137. A cut-off score of 51 has been proposed to identify the patients most at risk of sedentarism [32] and this value was used in the SPACE study for this purpose.

### Study objectives

The primary objectives of the SPACE study were to describe the prevalence and severity of early morning, day and night-time symptoms, as well as the patterns of physical activity, in stable COPD patients. The secondary objective of the study was to characterize the relationship between the 24-h symptoms and physical activity level and other COPD dimensions including, but not limited to, exacerbation history, severity of disease, comorbidities, level of dyspnea and health status.

### Sample size estimation

A sample size of 2000 patients offered a maximum margin of error (minimum precision) of 2% for estimating the percentage of patients within each category of the primary endpoint (symptoms and insufficient physical activity levels), considering maximum indetermination (*p* = 40%) and a confidence interval of 95%. Estimating that approximately 5% of patients would not be evaluable, due to missing data or not meeting all eligibility criteria, the final sample size was established at 2100 patients.

### Statistical analysis

All eligible patients who met all inclusion criteria and none of the exclusion criteria were included in full analysis set (FAS). All analyses were performed for FAS. Statistical analyses were of an explorative and descriptive nature. The differences in means or medians of continuous variables between two groups have been tested using the t-test for normally distributed variables, and Wilcoxon signed rank test for non-normally distributed variables. The mean differences between more than two groups were analyzed using one-way analysis of variance or Kruskal-Wallis Rank Sum test. The difference between the expected and the observed frequencies in one or more categories was assessed using the Chi-squared test. The relationship between two variables was assessed with the correlation analysis, using the Pearson or Spearman correlation coefficient, based on the distribution of variables. All statistical tests were two-sided and used a 5% significance level. All statistical analyses were performed using software R (https://www.r-project.org/), version 3.3.3.

## Results

### Patients’ characteristics

In total, 2190 patients were enrolled into the SPACE study. Of these, 28 patients did not fulfill all eligibility criteria and were excluded, with 2162 patients being included in the final analysis. Demographic and clinical characteristics of the study population are shown in Table 1. Two thirds of the patients were male, with a mean age of 66.9 years and a mean duration of COPD of 7.4 years. The demographics were broadly similar across countries, the mean age ranging from 65.1 years in Portugal to 69 years in Israel. In the FAS, 84.5% of patients had moderate or severe COPD, based on the severity of the airflow obstruction. At country level, this percentage varied from 77% in Portugal to 90% in Slovakia.

**Table 1:** Demographic and clinical characteristics of the study population

Characteristic	Patients (*N* = 2162)
Male sex, n (%)	1513 (70.0)
Age, mean (SD), years	66.9 (8.4)
BMI, mean (SD), kg/m^2^	27 (6.2)
Retired patients, n (%)	1755 (81.2)
High-school and university education, n (%) (*n* = 2157)	788 (36.4)
Active smoker, n (%)	691 (32.0)
Smoking history, mean (SD) pack years	41.31 (26.37)
Number of comorbidities, mean (SD)	1.28 (1.04)
% predicted FEV_1_, mean (SD)	52.6 (17.3)
Mean post-BD FVC (SD), mL (*n* = 2033)	2737.4 (846.5)
Mean post-BD FEV_1_/FVC (SD)	0.537 (0.105)
BODEx score, mean (SD)	2.6 (1.8)
COTE index, mean (SD)	1.5 (2.2)
Duration of COPD, mean (SD), years	7.4 (5.8)
COPD severity	
GOLD group I (mild)	142 (6.6)
GOLD group II (moderate)	1033 (47.8)
GOLD group III (severe)	793 (36.7)
GOLD group IV (very severe)	194 (9.0)
mMRC grade, median (Q1-Q3)	2 (1–3)
mMRC dyspnea grade, n (%)	
0	127 (5.9)
1	634 (29.3)
2	852 (39.4)
3	447 (20.7)
4	102 (4.7)
Total CAT score, mean (SD)	16.5 (7.9)
Exacerbations in the last year, n (%)	1335 (61.8)
Number of COPD exacerbations in the last year/patient, median (IQR)	1 (0–2)
Number of COPD-related visits in the last year/patient, median (IQR)	
To general practitioner (*n* = 1787)	2 (0–6)
To pulmonology / internal medicine specialist (*n* = 2145)	2 (2–4)

*BD* bronchodilator, *BMI* body mass index, *BODEx* Body mass index, Obstruction, Dyspnea, and Exacerbations, *CAT* COPD Assessment Test, *COTE* COPD specific comorbidity test, *FEV*_1_ Forced expiratory volume in 1 second, *FVC* Forced Vital Capacity, *GOLD* Global Initiative for Chronic Obstructive Lung Disease, *IQR* interquartile range, *mMRC* modified Medical Research Council, *SD* standard deviation

In total, 64.8% of patients had dyspnea of grade ≥ 2 as assessed by the mMRC scale. The following comorbidities were more frequent: confirmed CV disease 63.2%, hypertension 54.8%, confirmed diabetes 13.6%, anxiety 11% and confirmed depression 6.8%. Out of the total number of patients included in the SPACE study, 7.4% had a previous diagnosis of asthma.

Almost all patients (98.9%) were receiving COPD treatment at the time of enrollment and multiple patterns of treatments were identified (Additional file 1: Table S1).

### COPD symptoms (early morning, daytime & night-time)

Overall, 96.3% of patients reported at least one daytime (DT) symptom, 86.4% at least one early-morning (EM) symptom and 71.5% at least one night-time (NT) symptom (EM and NT symptoms were evaluated using the 6-item reporting). The prevalence of symptoms maintained a similar distribution and increased with COPD severity (based on airflow obstruction), with > 90% reporting symptoms in any part of the 24-h day, irrespective of COPD severity (Additional file 1 Table S2). The mean symptoms severity score (SD) was 11.33 (7.5) for DT, 1.14 (0.89) for EM and 0.76 (0.91) for NT symptoms (Additional file 1: Table S3).

**Fig. 1:**
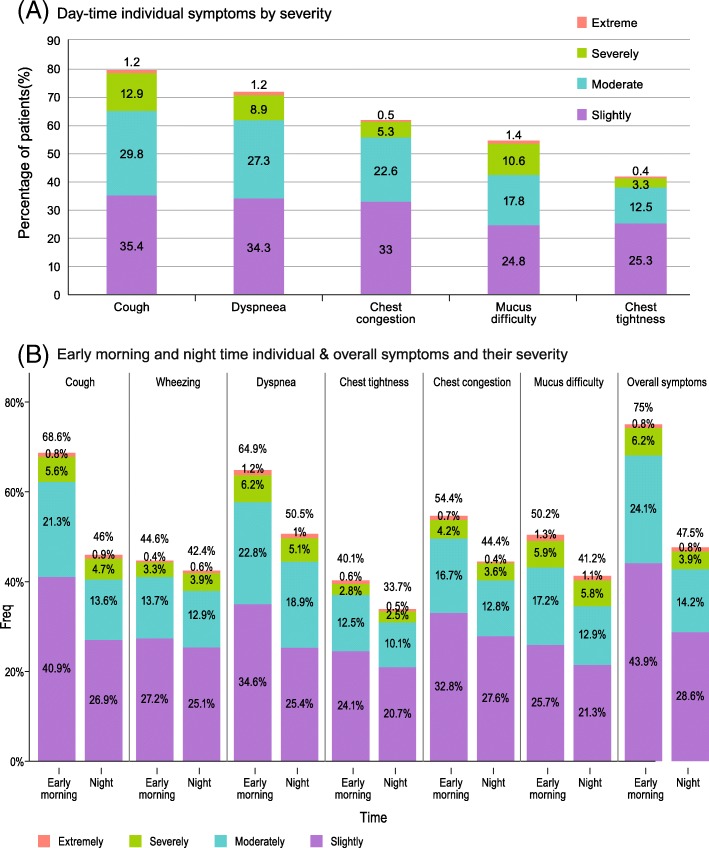
Prevalence of individual respiratory symptoms and their severity. **a** Day-time individual symptoms by severity. **b** Early morning and night time individual & overall symptoms and their severity

The prevalence of individual symptoms and their severity is presented in Fig. 1. In any part of the day, dyspnea and cough + sputum were reported by 80.2 and 78.16% of patients, respectively. In these two groups, the mean number of exacerbations (SD) in the previous year was significantly higher as compared to the group of patients without such symptoms: 3.17 (11.78) vs 1.12 (2.15) for patients with dyspnea, and 3.12 (11.80) vs 1.48 (4.16) for patients with cough + sputum (*p* < 0.001 for both).

A total of 65.2% patients reported tiredness during the day and 48% mentioned sleep disturbances in the night before the evaluation. Like DT symptoms, the impact of NT and EM symptoms was observed in 26.5% patients reporting night awakenings due to COPD symptoms, while 75% of patients mentioned limitations of their morning activities due to EM respiratory symptoms. A total of 19.2% patients reported the use of rescue medication during night-time and 31.1% in the early morning, with a mean number of puffs (SD) of 0.42 (1.09) in the night and 0.58 (1.02) in the morning.

A significant association between EM, DT and NT symptoms was described for each symptom combination (e.g., EM symptoms and DT symptoms, DT symptoms and NT symptoms, EM symptoms and NT symptoms; *p* < 0.001 for all). (Additional file 1: Table S4).

### Exacerbations

Overall, 1335 (61.8%) patients presented exacerbations in the previous year, with a mean number (SD) of any COPD exacerbations of 2.77 (10.63). More than half of patients presented mild and/or moderate exacerbations in the 12 months prior to enrollment. (Additional file 1: Table S5) However, the mean number of exacerbations (SD) in the previous year varied across countries from 0.64 (0.90) in Portugal to 6.34 (17.56) in Serbia.

### Physical activity level

Based on their own clinical judgement when assessing the physical activity level (PAL) of patients, the investigators estimated that 45.7% patients were active, 42.4% were insufficiently active and 11.9% were completely inactive. When the total minutes per week of moderate to vigorous physical activity were calculated based on the patients’ responses to EVS questions, the results showed that 32% considered themselves active, 35.1% insufficiently active and 32.9% completely inactive.

The assessment of the checklist with activities included in YPAS showed that the overall study population spent an average (SD) of 21.0 (18.1) hours/week performing physical activity with a total mean energy expenditure summary index (SD) of 4690.4 (4240.2) kcal/week. The mean summary index value (SD) was 42.9 (26.0), consisting of a vigorous index of 10.9 (14.6), a leisurely walking index of 16.0 (14.0), a moving index of 7.3 (3.6), a standing index of 6.3 (3.5) and a sitting index of 2.4 (1.1). Using the cut-off score of 51 in the YPAS summary index, a total of 68.4% of patients were identified as sedentary.

When stratifying patients by type of exacerbations in the previous year, PAL significantly decreased with increasing severity of exacerbations, as measured with YPAS (*p* < 0.001). (Additional file 1: Table S6) Similarly, when stratifying patients by number of comorbidities (0, 1 or ≥ 2), PAL significantly decreased with increasing number of comorbidities, as measured by both EVS and YPAS (*p* < 0.001). (Additional file 1: Table S7).

### Physical activity level by GOLD classes

**Fig. 2:**
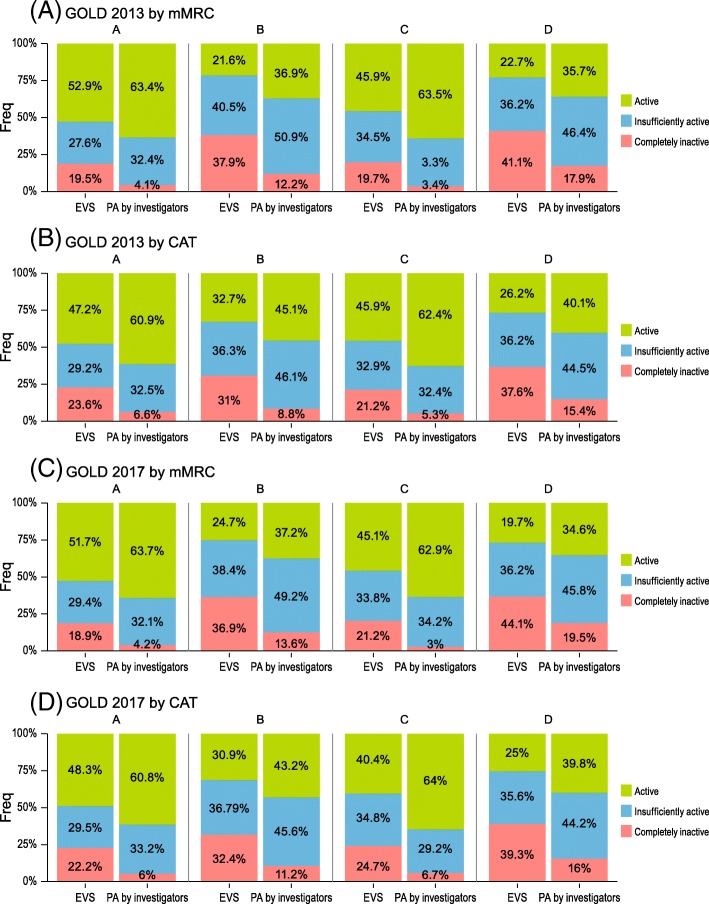
The distribution of physical activity level through GOLD classes by 2013 and 2017 editions. **a** GOLD 2013 by mMRC. **b** GOLD 2013 by CAT. **c** GOLD 2017 by mMRC. **d** GOLD 2017 by CAT

The distribution of physical activity level through GOLD classes by 2013 and 2017 editions is presented in Fig. 2. Groups A/C showed a higher PAL as compared with B/D, regardless of the questionnaire used in each GOLD edition (*p* < 0.001 for all) for both self-reported and investigators’ evaluations. Compared to the self-reports, the percentage of active patients was higher in the investigators’ evaluation, irrespective of the GOLD group. Around 20% of patients from group A considered themselves to be completely inactive, irrespective of the GOLD edition used.

Using the cut-off score of 51 in the YPAS summary index, the percentage of sedentary patients was > 50% in groups A/C and > 65% in groups B/C, varying by the questionnaire used for GOLD classification. (Additional file 1: Table S8).

### Correlations between symptoms, physical activity and other outcomes

There was a significant, strong correlation between symptoms in each part of the 24-h day and health status as assessed by CAT, but weaker with the severity of self-reported dyspnea as assessed with mMRC (*p* < 0.001 for all). (Additional file 1: Table S9) For self-reported physical activity as assessed by EVS, the correlations with CAT or mMRC were significant, but weak (p < 0.001). (Additional file 1: Table S10).

The analysis of the relationship between symptoms in each part of the 24-h day and PAL indicated negative, weak correlations between symptoms and patient-reported physical activity assessed with either EVS or YPAS (p < 0.001 for both). (Additional file 1: Table S11).

Similarly, for dyspnea, irrespective of the severity, weak, negative correlations with PAL were found for the PA evaluated by investigators or YPAS (*p* < 0.001 for both). (Additional file 1: Table S12) Although significant (*p* < 0.001), the correlation between EM, DT and NT symptoms and exacerbation history was weak. Negative correlations were found between comorbidities and PAL and between sleep disturbances and PAL as assessed with YPAS (*p* < 0.001).

## Discussion

This observational, cross-sectional study showed that despite regular treatment, the majority of stable COPD patients with moderate to severe disease continue to experience daily variable symptoms and to have a sedentary lifestyle. The percentage of inactive patients is high in any GOLD ABCD groups, a result consistent throughout each PA questionnaire used in the SPACE study. Comprehensively assessing 24-h respiratory symptoms and physical activity level in a large multinational cohort of COPD patients, our study found a modest, but consistent, correlation between physical activity level and symptoms. Although the self-reported overall physical activity level of patients was low, it nevertheless appeared to be overestimated by their physicians.

Assessing respiratory symptoms over 24 h, a high percentage of stable patients from our real-life cohort reported mild and moderate symptoms throughout the day, despite regular treatment. In line with other studies performed in a similar population of patients, our results indicate that symptom variability is higher than expected in stable COPD patients [7, 8, 12]. Future prospective studies are needed to explore how symptoms change from early morning to daytime and night-time, in terms of individual symptom type and severity, and if any patterns can be identified to guide and tailor the treatment of patients.

The second component of the SPACE study co-primary objective was physical activity in stable COPD patients; this concept was evaluated in a non-interventional manner from both the patients’ and physicians’ perspective, using interviewer- and self-reported questionnaires and clinical judgement, respectively. YPAS, a validated tool in elderly subjects, which captures a broad spectrum of low-intensity activities usually not included in other questionnaires, was used to evaluate PAL [31]. YPAS results indicated that almost two-thirds of patients in the SPACE study were sedentary, with less time spent in performing activities (approx. 3 h/day) compared to healthy subjects of the same age or older (approx. 4–9 h/day) [31, 33]. These results are similar to those reported in the study which validates the YPAS in COPD [32]. Apart from a shorter duration of activity, the study participants tended to spend a longer time not performing any activity, as reflected by the sitting and standing index scores, a result which is in line with previous papers on this topic [34, 35]. Sedentary time and PA were recently found to be important risk factors for hospitalization in COPD patients [36]. Additionally, a longer sitting time seems to correlate with an increased risk of mortality, independent of leisure time physical activity undertaken [37]. Thus, clinicians should be encouraged to investigate the patterns of inactivity in COPD patients and educate inactive individuals with regard to interrupting their sitting times [20, 38, 39].

To our knowledge, no other studies have investigated the physicians’ perception of physical activity in their COPD patients, nor compared it with patients’ self-reported physical activity. The SPACE study investigators were asked to use clinical judgement in assessing the level of physical activity of their patients, leaving at their discretion how to explore it. During the clinical interview at the study visit, patients answered two questions from the EVS program [30] to report their PAL, but the eCRF did not disclose patients’ answers to investigators. Physicians overestimated the level of physical activity in this population of stable, yet sedentary, COPD patients. Besides a different perception of the concept of physical activity, the difference between physicians and patients in estimating PALs indicates that patients are less active than their physicians perceive them to be, that physicians do not commonly assess PAL in respiratory patients and lack the tools to do so. These results are an indirect indicator of how physicians may underestimate the consequences of the physical inactivity in their COPD patients. As the fourth leading risk factor for global mortality worldwide [40], physical inactivity should be aggressively fought. A large prospective study [41] has found that the introduction of lower levels of activity, such as 15 min walking 6 days per week, might be beneficial in reducing all-cause mortality. These results are more realistic for COPD patients, in whom replacing sedentary behavior with light activities would be much more feasible than increasing the time spent in moderate to vigorous activities [20, 39, 42]. Nevertheless, all these findings serve to underline the unmet need for a set of standardized tools and guidance for physicians to evaluate, promote and monitor physical activity in COPD patients in everyday clinical practice.

Understanding the relationship between respiratory symptoms across the 24-h day and physical activity has the potential to provide valuable insights for the clinical management of COPD in the real-life setting. However, there is no well-defined approach to evaluating symptoms or physical activity level and, in general, these clinical determinants are not assessed together. Our results indicated significant, but weak, relationships between 24-h symptoms and physical activity, which is not allowing us to draw definite conclusions. The data about this relationship in the literature are divergent and it is not yet known which component of the symptoms is related to physical activity. Previous research indicated the existence of a significant association between COPD symptoms and physical activity [4, 7], but no causality was proven. In the SPACE study, we used three different instruments to assess physical activity and the results were consistent across all of these. Moreover, other studies showed that dyspnea, for example, is not the main factor limiting exercise in individuals with COPD [34] and many patients chose to reduce their physical activity level rather than being restricted by symptoms [17, 43, 44]. One important finding from our study was that PAL decreased with increasing severity of exacerbations. Exacerbations cause not only worsening of symptoms, but also affect patients’ daily activities, while inactive patients tend to have more severe exacerbations compared to active patients [45, 46]. Furthermore, PAL decreased with an increasing number of comorbidities. Data in the literature indicate that daily PAL in COPD patients is impaired by comorbidities, irrespective of their type and independent of the level of airflow obstruction [47]. These findings show that other dimensions of physical activity such as comorbidities, environmental factors and previous history of physical activity [44] should be explored, combined with symptoms, to better understand and address the low physical activity level in COPD patients. Modifying complex health behavior, such as increasing levels of physical activity, is a difficult task and requires a multidisciplinary approach [42]. More studies exploring the multimodal interventions (pharmacological and behavioral) [48], more specific recommendations for increasing PA in people with COPD [17, 20] and, perhaps, a change in the mindset of healthcare professionals to adequately understand physical activity levels in their patients, are needed in order to guide clinicians in current practice.

One of the main findings of our study is that the percentage of sedentary patients in mild stages of COPD is higher than that anticipated by investigators, as shown by their clinical judgement, irrespective of the GOLD edition used. Primarily, this result supports previous findings showing an increased proportion of patients with low PAL even in mild stages of COPD [38, 49–51]. Secondly, while patients tend to consider themselves more inactive, physicians tend to overestimate the PAL in each GOLD group. Importantly, the YPAS results indicate that more than half of stable COPD patients in our cohort were sedentary, irrespective of GOLD group. Previous studies showed that COPD patients experience a faster decline in PAL over time compared to healthy people [52]. This observation and the constant association between PA and mortality emphasize the importance of a proactive screening of physical inactivity in COPD patients from earlier stages of disease. Maybe physical inactivity is indeed the “missing link” in understanding the progression of COPD [53]. Further insights in PA patterns and clinical characteristics of patients would allow for a more tailored approach, adapted to their needs and living conditions. In this context, it seems reasonable to challenge the current paradigm of assessing and treating COPD and to consider physical activity level as one of the determinants of the GOLD ABCD groups, as well as including an increase of PAL in patients’ written action plans as an objective in disease management.

A strength of this study is the comprehensive assessment of respiratory symptoms and PAL using multiple, validated questionnaires. Furthermore, we believe that this large population of patients is representative of the stable COPD populations, since patients were enrolled in multiple countries from various healthcare settings, including public and private practices and hospitals. However, several limitations of this study should be mentioned and one is the fact that we cannot provide information about any causal relationship, as SPACE was a real-world evidence study with a cross-sectional design. Specific relationships between symptoms in any part of the 24-h day, physical activity and other outcomes should be explored in more depth, adjusting for confounding factors. In the SPACE study, patient-reported outcomes (PROs) were collected through printed versions of the questionnaires and were subject to missing data, as there was no mechanism to prevent omitting questions. Although the electronic PROs are attractive, their use is not yet implemented in current clinical practice in all participating countries, thus having an interventional character. Lastly, there are no standardized tools for assessing physical activity levels. In our study, physical activity was measured only subjectively, through interviews using tools validated in COPD patients (YPAS). Self-reported PA is often subject to recall bias, lacks precision and, in many cases, does not correlate well with objectively measured physical activity [41]. However, even in this context, our results indicate a real need for educating clinicians to adequately evaluate and monitor physical activity levels in clinical practice and the introduction of validated, standardized questionnaires might be the simplest way of achieving this.

## Conclusions

Despite regular treatment, most stable outpatients with COPD from the SPACE study still presented mild to moderate symptoms, irrespective of disease severity. In any GOLD group, the percentage of inactive patients was high. While physical activity levels were low in this large multinational COPD cohort, they were, nevertheless, overestimated by physicians. With recent evidence pointing to the negative consequences of inactivity, maybe physical activity level plays a bigger role in profiling patients than previously thought and, therefore, its integration into current ABCD GOLD groups would better guide clinicians in making effective treatment decisions. Adequate tools to screen and monitor sedentarism patterns and a more active promotion of physical activity among COPD patients are urgently needed to ensure long-term health benefits.

## Additional files

Additional file 1: **Table S1**. Main COPD treatment patterns by therapeutic classes, irrespective of other maintenance treatments or short-acting bronchodilators. **Table S2.** Prevalence of any 24-h and specific respiratory symptoms by COPD severity in FAS**. Table S3**. Daytime, night-time and early morning symptoms severity in FAS. **Table S4**. The relationship between early morning, daytime and night-time symptoms. **Table S5**. Distribution of patients and mean number of events by type of exacerbations. **Table S6**. PA characteristics (YPAS) in patients with exacerbations in the previous year. **Table S7**. Physical activity parameters by the number of comorbidities. **Table S8**. Distribution of patients with YPAS total score < 51 in each GOLD group using 2013 and 2017 edition. **Table S9**. Correlation between 24-h symptom severity scores and patient-reported outcomes. **Table S10**. Correlation between physical activity level (EVS) and other variables. **Table S11**. Correlations between symptoms in each part of the day and PAL (EVS and YPAS). **Table S12**. Correlations between physical activity and severity of dyspnea. **Table S13**. Daytime, night-time and early morning symptom severity in selected subpopulations based on different treatment patterns (DOCX 63 kb)

Additional file 2: SPACE study – list of Investigators (DOCX 121 kb)

## Abbreviations

BD: Bronchodilator
BODEx: Body mass index, obstruction, dyspnea and exacerbations
CAT: COPD Assessment Test
COPD: Chronic Obstructive Pulmonary Disease
COTE: COPD specific comorbidities test
CRF: Case Report Form
DT: Day-time
EM: Early morning
EMSCI: Early Morning Symptoms of COPD Instrument
E-RS™: COPD: Evaluating Respiratory Symptoms in COPD
EVS: Exercise vital sign
FAS: Full Analysis Set
FEV_1_: Forced expiratory volume in 1 s
FVC: Forced vital capacity
GOLD: Global initiative for chronic Obstructive Lung Disease
ICS: Inhaled corticosteroid
IQR: Interquartile range
LABA: Long-acting β_2_-agonist
LAMA: Long-acting muscarinic antagonist
mMRC: Modified Medical Research Council
NiSCI: Night-time Symptoms of COPD Instrument
NT: Night time
SD: Standard deviation
SGC: Systemic glucocorticoids
YPAS: Yale Physical Activity Survey
