# Supplementary material for: Importance of the relationship between symptoms and self-reported physical activity level in stable COPD based on the results from the SPACE study
Source: Respir Res. 2019 May 14;20:89. doi: 10.1186/s12931-019-1053-7 (PMC6518503; doi:10.1186/s12931-019-1053-7)
Supplement: Supplementary file 1 — Table S1. Main COPD treatment patterns by therapeutic classes, irrespective of other maintenance treatments or short-acting bronchodilators. Table S2. Prevalence of any 24-h and specific respiratory symptoms by COPD severity in FAS. Table S3. Daytime, night-time and early morning symptoms severity in FAS. Table S4. The relationship between early morning, daytime and night-time symptoms. Table S5. Distribution of patients and mean number of events by type of exacerbations. Table S6. PA characteristics (YPAS) in patients with exacerbations in the previous year. Table S7. Physical activity parameters by the number of comorbidities. Table S8. Distribution of patients with YPAS total score < 51 in each GOLD group using 2013 and 2017 edition. Table S9. Correlation between 24-h symptom severity scores and patient-reported outcomes. Table S10. Correlation between physical activity level (EVS) and other variables. Table S11. Correlations between symptoms in each part of the day and PAL (EVS and YPAS). Table S12. Correlations between physical activity and severity of dyspnea. Table S13. Daytime, night-time and early morning symptom severity in selected subpopulations based on different treatment patterns (DOCX 63 kb) [file 12931_2019_1053_MOESM1_ESM.docx]

Table A1. Main COPD treatment patterns by therapeutic classes, irrespective of other maintenance treatments or short-acting bronchodilators

| **Treatment pattern, n (%)** | **N=2162** |
| --- | --- |
| LABA/LAMA/ICS | 549 (25.4) |
| LABA/LAMA/ICS + any other combination | 336 (15.5) |
| LABA/ICS | 307 (14.2) |
| LABA/ICS + any other combination | 112 (5.2) |
| LABA/LAMA | 411 (19.0) |
| LABA/LAMA + any other combination | 77 (3.6) |
| LAMA | 173 (8.0) |
| LAMA + any other combination | 35 (1.6) |
| LABA | 78 (3.6) |
| LABA + any other combination | 11 (0.5) |
| ICS | 15 (0.7) |
| ICS + any other combination | 19 (0.9) |
| Systemic glucocorticoids | 54 (2.49) |

Any other combination refers to any other combination between long-acting bronchodilators and ICS not mentioned above (e.g., LAMA/ICS), as well as any combinations of non-bronchodilators or non-ICS (e.g., methylxanthines, mucolytic agents, leukotriene receptor antagonists, phosphodiesterase-4 inhibitors, oxygen or systemic corticoids).

ICS=inhaled corticosteroid; LABA=long-acting β_2_-agonist; LAMA=long-acting muscarinic antagonist.

Table A2. Prevalence of any 24-hour and specific respiratory symptoms by COPD severity in FAS

| **Symptoms** | **GOLD severity** | | | |
| --- | --- | --- | --- | --- |
|  | **GOLD I** (n=142) | **GOLD II** (n=1033) | **GOLD III**  (n=793) | **GOLD IV**  (n=194) |
| Any 24- hour symptoms | 130 (91.55) | 995 (96.32) | 780 (98.36) | 193 (99.48) |
| Early-morning | 109 (76.76) | 880 (85.19) | 701 (88.40) | 177 (91.24) |
| Night | 87 (61.27) | 708 (68.54) | 597 (75.28) | 153 (78.87) |
| Daytime | 127 (89.44) | 987 (95.55) | 776 (97.86) | 192 (98.97) |

********P-value was <0.001 for any time using Pearson's Chi-squared.*

Table A3. Daytime, night-time and early morning symptoms severity in FAS

| **Symptoms** | **N=2162** |
| --- | --- |
| Daytime* |  |
| RS-Chest symptoms, mean (SD) (n=2150) | 2.31 (2.29) |
| RS-Cough & Sputum, mean (SD) (n=2146) | 3.14 (2.25) |
| RS-Breathlessness, mean (SD) (n=2143) | 5.88 (4.27) |
| RS-Total, mean (SD) (n=2100) | 11.33 (7.50) |
| Night-time** |  |
| NiSCI 6-Item Symptom Summary Score, mean (SD) (n=2134) | 0.67 (0.73) |
| NiSCI Overall COPD Severity Score, mean (SD) (n=2157) | 0.72 (0.91) |
| Early morning** |  |
| EMSCI 6-Item Symptom Summary Score, mean (SD) (n=2137) | 0.83 (0.72) |
| EMSCI Overall COPD Severity Score, mean (SD) (n=2153) | 1.14 (0.89) |

The correlation between the 6-item severity score and overall COPD severity score for both NiSCI and EMSCI was strong (*r*=0.77, p<0.001 for both)

* Mean scores (SD) based on one single assessment on the day of the study visit; Ranges: RS-Chest symptoms: 0 to 12; RS-Cough & Sputum: 0 to 11; RS-Breathlessness: 0 to 17; RS-Total: 0 to 40; higher scores = more severe symptoms

** Mean scores (SD) based on one single assessment on the day of the study visit; Ranges for both EMSCI & NiSCI: 0 to 4; higher scores = more severe symptoms

EMSCI=Early Morning Symptoms of COPD Instrument; FAS=full analysis set; NiSCI=Night-time Symptoms of COPD Instrument; RS=respiratory symptoms.

Table A4. The relationship between early morning, daytime and night-time symptoms

|  | **Early-morning symptoms** | **Night-time symptoms** | **Daytime symptoms** |
| --- | --- | --- | --- |
| Early-morning symptoms (using RS-Total)* | 1 | 0.8 | 0.82 |
| Night-time 6-Item symptoms* | 0.8 | 1 | 0.72 |
| Daytime 6-Item symptoms* | 0.82 | 0.72 | 1 |

**Pearson correlation with P<0.001*

RS=respiratory symptoms.

Table A5. Distribution of patients and mean number of events by type of exacerbations

| **Characteristics** | **N=2162** |
| --- | --- |
| Mild exacerbations, n (%) | 743 (34.37) |
| Number of mild exacerbations, mean (SD), median (Q1, Q3) | 1.93 (10.39),  0 (0,1) |
| Moderate exacerbations | 794 (36.73) |
| Number of moderate exacerbations, mean (SD), median (Q1, Q3) | 0.61 (1.15),  0 (0,1) |
| Severe exacerbations | 326 (15.08) |
| Number of severe exacerbations, mean (SD), median (Q1, Q3) | 0.22 (0.64),  0(0,0) |

SD=standard deviation; Q=quartile.

Table A6. PA characteristics (YPAS) in patients with exacerbations in the previous year

|  | **Exacerbation history in the previous year** | | | |
| --- | --- | --- | --- | --- |
|  | No exacerbations  (n=827) | Mild exacerbations (n=398) | Moderate exacerbations (n=611) | Severe exacerbations (n=326) |
| YPAS checklist total time (min/week), median (IQR) | 1210.38 (1053.57) | 1383.49 (1051.78) | 1315.44 (1122.58) | 1139.20 (1121.51) |
| Energy expenditure summary index (Kcal/week), median (IQR) | 4606.23 (4197.71) | 5152.49 (4225.35) | 4816.03 (4276.48) | 4102.21 (4238.21) |
| YPAS total score, median (IQR) | 45.81 (26.42) | 45.42 (25.87) | 42.02 (25.91) | 33.75 (22.51) |

Except YPAS checklist total time between patients with TT compared to patients without TT where p=0.001, for all the other parameters evaluated between groups, p<0.001

IQR=interquartile range; PA=physical activity; TT=triple therapy; YPAS=Yale Physical Activity Survey.

Table A7. Physical activity parameters by the number of comorbidities

|  | **Number of comorbidities** | | | **P-value** |
| --- | --- | --- | --- | --- |
|  | **0** | **1** | **≥2** |  |
| Patient number | 503 | 879 | 780 |  |
| EVS index [(mean (SD)] | 1.12 (0.80) | 1.00 (0.80) | 0.90 (0.80) | <0.001 |
| YPAS total score  [(mean (SD)] | 46.84 (26.11) | 44.42 (26.53) | 38.50 (24.53) | <0.001 |

EVS index was coded into numeric classes and means calculated based on the 3 categories of “Total minutes per week of moderate or vigorous physical activity”, as following:

- Completely inactive (0 min/week) = 0
- Insufficiently active (1–149 min/week) = 1
- Active, meeting national physical activity recommendations (≥150 min/week) = 2

Table A8. Distribution of patients with YPAS total score <51 in each GOLD group using 2013 and 2017 edition

| **ABCD group** | **GOLD 2013 mMRC** | **GOLD 2017 mMRC** | **GOLD 2013 CAT** | **GOLD 2017 CAT** |
| --- | --- | --- | --- | --- |
| Group A | 211 (51.5) | 282 (53.8) | 148 (54.6) | 190 (54.0) |
| Group B | 283 (72.0) | 543 (70.9) | 346 (65.0) | 635 (67.7) |
| Group C | 214 (61.0) | 143 (60.3) | 95 (55.9) | 53 (59.6) |
| Group D | 770 (76.4) | 510 (80.3) | 889 (74.8) | 600 (76.6) |

*P-value <0.001 for all; P-value was calculated using Pearson’s Chi squared test*

CAT=COPD Assessment Test; GOLD=Global Initiative for Chronic Obstructive Lung Disease; mMRC= modified Medical Research Council.

Table A9. Correlation between 24-hour symptom severity scores and patient-reported outcomes

|  | **Early-morning symptoms** | **P-value** | **Night-time symptoms** | **P-value** | **Daytime symptoms** | **P-value** |
| --- | --- | --- | --- | --- | --- | --- |
| **CAT** | **0.68** | **<0.001** | **0.62** | **<0.001** | **0.74** | **<0.001** |
| Comorbidities (COTE) | 0.03 | 0.188 | 0.03 | 0.198 | 0.07 | 0.001 |
| BODEx | 0.32 | <0.001 | 0.28 | <0.001 | 0.43 | <0.001 |
| **mMRC** | **0.35** | **<0.001** | **0.32** | **<0.001** | **0.47** | **<0.001** |
| Post-BD FEV_1_% predicted | -0.21 | <0.001 | -0.17 | <0.001 | -0.28 | <0.001 |
| Post-BD FEV_1_/FVC % | -0.06 | 0.003 | -0.03 | 0.14 | -0.12 | <0.001 |
| Number of severe exacerbation | 0.14 | <0.001 | 0.11 | <0.001 | 0.15 | <0.001 |
| Healthcare resource utilization (number of COPD-related visits) | 0.09 | <0.001 | 0.06 | 0.009 | 0.07 | 0.002 |
| Adherence to respiratory medication | -0.008 | 0.704 | -0.0007 | 0.976 | -0.014 | 0.535 |
| Exacerbation history | 0.19 | <0.001 | 0.18 | <0.001 | 0.2 | <0.001 |

The Pearson’s product-moment correlation was used.

BD=bronchodilator; BODEx=body mass index, obstruction, dyspnea and exacerbations; CAT=COPD Assessment Test; COPD=Chronic Obstructive Pulmonary Disease; COTE=COPD specific comorbidities test; FEV_1_/FVC=forced expiratory volume in 1 second/forced vital capacity; mMRC=modified Medical Research Council.

Table A10. Correlation between physical activity level (EVS) and other variables

|  | **Pearson correlation *r*** | **P value** |
| --- | --- | --- |
| CAT | -0.23 | <0.001 |
| mMRC | -0.36 | <0.001 |

CAT=COPD Assessment Test; EVS=exercise vital sign; mMRC=modified Medical Research Council.

Table A11. Correlations between symptoms in each part of the day and PAL (EVS and YPAS)

|  | **Early-morning symptoms** | **Night-time symptoms** | **Daytime symptoms** |
| --- | --- | --- | --- |
| EVS* | -0.14 | -0.13 | -0.2 |
| YPAS total score (summary index of physical activity)* | -0.14 | -0.13 | -0.24 |
| YPAS checklist total time (min/week)^#^ | -0.06 | -0.07 | -0.027 |
| Energy expenditure summary index (Kcal/week)^#^ | -0.07 | -0.07 | -0.12 |

**Pearson correlation with P<0.001; ^#^Pearson correlation with P<0.01 except for the correlation between YPAS checklist total time and daytime symptom severity (P=0.21)*

EVS=exercise vital sign; min=minutes; kcal=kilocalories; PAL=physical activity level; YPAS=Yale Physical Activity Survey.

Table A12. Correlations between physical activity and severity of dyspnea

|  | **Average severity of dyspnea during 24 hours** | | **Maximum severity of dyspnea during 24 hours** | |
| --- | --- | --- | --- | --- |
|  | Correlation | P value | Correlation | P value |
| Patient activity level reported by investigator | -0.23 | <0.001 | -0.23 | <0.001 |
| EVS index | -0.21 | <0.001 | -0.19 | <0.001 |
| YPAS checklist total time | -0.13 | <0.001 | -0.13 | <0.001 |
| Energy expenditure summary index | -0.14 | <0.001 | -0.15 | <0.001 |
| YPAS total score | -0.22 | <0.001 | -0.22 | <0.001 |

*The Pearson’s product-moment correlation was used.*

EVS=exercise vital sign; YPAS=Yale Physical Activity Survey.

**Study results in selected subpopulations based on different treatment patterns**

***Daytime, night-time and early morning symptoms***

We have performed a subgroup analysis for patients receiving LAMA/LABA/ICS, irrespective of the rescue medication or other maintenance medication (e.g., non-bronchodilator or non-ICS). The patients receiving triple therapy (n=885) presented significantly higher mean scores of severity for early morning, daytime and night-time compared to patients receiving any other treatment, but not triple therapy (n=1277), especially in RS-Total [mean RS-Total (SD): 12.44 (7.80) vs10.56 (7.19), p<0.001 for all scores]. For other treatment patterns, significant differences were seen for all symptom scores in the LABA/LAMA group and for individual RS-scores (chest symptoms and cough & sputum) in the LABA/ICS group (p<0.001). (Table A14)

Table A13. Daytime, night-time and early morning symptom severity in selected subpopulations based on different treatment patterns

| **Specific symptom score** | **Specific subgroups** | |
| --- | --- | --- |
|  | **LAMA only**  **(n=197)** | **No LAMA only**  **(n=1965)** |
| RS-total* [(mean (SD)] | 8.92 (6.64) | 11.57 (7.54) |
|  | **LABA/LAMA**  **(n=488)** | **No LABA/LAMA**  **(n=1674)** |
| EMSCI score** [(mean (SD)] | 0.70 (0.66) | 0.87 (0.74) |
| NiSCI score** [(mean (SD)] | 0.56 (0.68) | 0.71 (0.75) |
| RS-Total* [(mean (SD)] | 10.04 (6.94) | 11.71 (7.61) |
| RS-Chest symptoms* [(mean (SD)] | 1.87 (1.95) | 2.44 (2.36) |
|  | **LABA/ICS**  **(n=419)** | **No LABA/ICS**  **(n=1743)** |
| RS-Chest symptoms* [(mean (SD)] | 2.67 (2.32) | 2.22 (2.27) |
| RS-Cough & Sputum* [(mean (SD)] | 3.48 (2.18) | 3.06 (2.26) |
|  | **LABA/LAMA/ICS**  **(n=885)** | **No LABA/LAMA/ICS**  **(n=1277)** |
| EMSCI score** [(mean (SD)] | 0.91 (0.77) | 0.78 (0.68) |
| NiSCI score** [(mean (SD)] | 0.75 (0.77) | 0.62 (0.70) |
| RS-Total* [(mean (SD)] | 12.44 (7.80) | 10.56 (7.19) |
| RS-Breathlessness* [(mean (SD)] | 6.70 (4.41) | 5.32 (4.08) |

This table presents only statistically significant results as defined by the p-value <0.001 calculated with the Mann-Whitney test.

The LAMA only group includes patients receiving LAMA only and LAMA only + non-ICS therapies.

*Mean scores (SD) based on one single assessment on the day of the study visit; Ranges: RS-Chest symptoms: 0 to 12; RS-Cough & Sputum: 0 to 11; RS-Breathlessness: 0 to 17; RS-Total: 0 to 40; higher scores = more severe symptoms.

**Mean scores (SD) based on one single assessment on the day of the study visit; Ranges for both EMSCI & NiSCI: 0 to 4; higher scores = more severe symptoms.

EMSCI=Early Morning Symptoms of COPD Instrument; FAS=full analysis set; ICS=inhaled corticosteroid; LABA=long-acting β_2_-agonist; LAMA=long-acting muscarinic antagonist; NiSCI=Night-time Symptoms of COPD Instrument; RS=respiratory symptoms.

***Exacerbations***

The group receiving triple therapy presented a higher percentage of patients with exacerbations in the previous 12 months (68.4%) and a higher mean number (±SD) of severe exacerbations [0.30 (0.79)] compared with the patients not receiving triple therapy [57.2% and 0.16 (0.51), respectively] (p<0.001 for all).

***Physical activity level***

The group of patients with triple therapy (LAMA/LABA/ICS) had a significantly lower physical activity level compared to the group of patients not receiving triple therapy, as measured with YPAS (Table A14). For the other combined therapies, no significant differences were found.

Table A14. PA characteristics (YPAS) in triple therapy group

|  | **TT** | |
| --- | --- | --- |
|  | **TT**  **(n=885)** | **No TT**  **(n=1277)** |
| YPAS checklist total time (min/week), median (IQR) | 1188.62 (1072.61) | 1311.51 (1092.63) |
| Energy expenditure summary index (Kcal/week), median (IQR) | 4402.70 (4250.88) | 4889.72 (4222.98) |
| YPAS total score, median (IQR) | 40.32 (25.90) | 44.60 (25.83) |

*Except YPAS checklist total time between patients with TT compared to patients without TT, where p=0.001. For all the other parameters evaluated between groups, p<0.001.*

Triple therapy consisted of LABA/LAMA/ICS, irrespective of the rescue medication or other maintenance medication (e.g., non-bronchodilator or non-ICS).

ICS=inhaled corticosteroid; IQR=interquartile range; LABA=long-acting β_2_-agonist, LAMA=long-acting muscarinic antagonist; PA=physical activity; TT=triple therapy; YPAS=Yale Physical Activity Survey.
