# Supplementary material for: Importance of the relationship between symptoms and self-reported physical activity level in stable COPD based on the results from the SPACE study
Source: Respir Res. 2019 May 14;20:89. doi: 10.1186/s12931-019-1053-7 (PMC6518503; doi:10.1186/s12931-019-1053-7)
Supplement: Supplementary file 2 — SPACE study – list of Investigators (DOCX 121 kb) [file 12931_2019_1053_MOESM2_ESM.docx]

**SPACE study – list of Investigators**

**Austria**: Bernd Lamprecht, Ralf Harun Zwick, Lea Sator, Robert Voves, Joze Messner, Gerhard Köberl, Andrea Keckeis, Michael Muntean, Peter H. Heininger

**Belgium**: Capiau Luc, Corhay Jean-Louis, Etienne Isabelle, Fiévet Frédéric, Haenebalcke Christel, Vereecken Guy, Vincken Walter

**Bulgaria**: Palaveev Kiril, Angelova Sofia, Haytova-Vasileva Nezabravka, Krancheva-Kadeva Vanya, Petrova Galina, Vasileva Violina, Nikolova Penka, Alexandrova Radka, Draganova-Konakchieva Stefka, Halvadzhieva Neli, Kichukova Stanka, Marinova Krastina, Pavlova Sashka, Ruseva Nina, Stoychev Sivilyan, Arabadzhiev Damyan, Dacheva Yordanka, Georgieva Rumyana, Marinova Yordanka, Mihaylov Svetlan, Shtereva Emiliya

**Greece**: Kontakiotis Theodore, Tsoukalas Georgios, Kainis Ilias, Konstantinidis Athanasios, Terrovitou Chrysavgi, Evaggelopoulou Efstathia, Manos Emmanouil, Georgopoulos Dimitrios

**Israel**: Adir Yochai, Bar-Shai Amir, Fink Gershon, Izbicki Gabriel, Schwarz Yehuda, Kremer Mordechai

**The Netherlands**: Bresser Paul, de Jong Wouter, Lunde Ragnar, de Vries Michiel, van Lanen Marc, Boersma Wim, Broeders Marielle

**Poland**: Kołodyńska-Jeridi Justyna, Kazjaka-Olszewska Anna, Michnar Marek

Kubina Elżbieta, Markiewicz-Bendkowska Irena, Lubarska Maria, Barbara Janszarek, Kremer Danuta, Tęsiorowska Joanna, Kuczyńska Kinga, Cieślak Małgorzata, Barela-Malczewska Anna, Bokiej Juliusz, Szymon Dworniczak, Hajoł Elżbieta, Tsekov Małgorzata Lisowska Barbara, Kot Agata, Kisała Aleksandra

**Portugal**: Pires Nuno, Guimarães Maria, Guimarães Miguel, Araújo David, Ferreira Jorge, Cordeiro Carlos, Rodrigues Cidália, Pinto Paula, Cardoso João, Costa Rui, André Sandra, Bugalho António, Sousa Susana Brito Ulisses, Munhá João

**Romania**: Arghir Oana, Boisteanu Daniela, Dumitrache Rujinski Stefan, Fira-Mladinescu Ovidiu, Gheorghiu-Brinaru Manuela, Man Milena, Mincu Viorica, Miron Ramona, Nicolosu Dragos, Olar Emilia, Postolache Paraschiva, Rajnoveanu Ruxandra, Todea Doina, Teoibas Serban Carmen, Topana Iuliana, Ungureanu Dragos, Vancea Dorin

**Serbia**: Timotijevic Ljiljana, Dimic Dejan, Zdravkovic-Mihajlovic Aleksandra, Nikcevic Ljiljana, Nikolic Brstina Ivana, Radulovic Vesna, Vukadinovic Nerandzic Sofija, Orlovic Smiljana, Menkovic Jelena, Uletilovic Tanja, Mikavika Ivana, Zujovic Dejan, Filipovic Milos, Petrov Valentin, Mirkovic Ljiljana, Blagojevic Nadica, Trninic Stankovic Milena, Stefanovic Bratislav, Jovanovic Sasa, Jankovic Goran, Ilic Miroslav, Somborac Stevan, Jankov Jelena, Balog Klajn Marta, Andrijevic Ana, Ristic Zorica, Jokic Tatjana, Miloskovic Vladana, Urukalo Svetlana

**Slovakia**: Kavkova Denisa, Kurthova Svetlana, Frajtova Luboslava, Suchanova Erika, Arpasova Katarina, Hrinova Maria, Jurco Peter, Zacik Miroslav, Plutinsky Jan , Golubov Alexander
